# A Tale of Two Maladies? Pathogenesis of Depression with and without the Huntington’s Disease Gene Mutation

**DOI:** 10.3389/fneur.2013.00081

**Published:** 2013-07-09

**Authors:** Xin Du, Terence Y. C. Pang, Anthony J. Hannan

**Affiliations:** ^1^Behavioural Neuroscience Division, Florey Institute of Neuroscience and Mental Health, University of Melbourne, Parkville, VIC, Australia; ^2^Department of Anatomy and Neuroscience, University of Melbourne, Parkville, VIC, Australia

**Keywords:** Huntington’s disease, neurodegeneration, depression, psychiatric disorders, serotonin, BDNF, stress, polyglutamine disease

## Abstract

Huntington’s disease (HD) is an autosomal dominant disorder caused by a tandem repeat expansion encoding an expanded tract of glutamines in the huntingtin protein. HD is progressive and manifests as psychiatric symptoms (including depression), cognitive deficits (culminating in dementia), and motor abnormalities (including chorea). Having reached the twentieth anniversary of the discovery of the “genetic stutter” which causes HD, we still lack sophisticated insight into why so many HD patients exhibit affective disorders such as depression at very early stages, prior to overt appearance of motor deficits. In this review, we will focus on depression as the major psychiatric manifestation of HD, discuss potential mechanisms of pathogenesis identified from animal models, and compare depression in HD patients with that of the wider gene-negative population. The discovery of depressive-like behaviors as well as cellular and molecular correlates of depression in transgenic HD mice has added strong support to the hypothesis that the HD mutation adds significantly to the genetic load for depression. A key question is whether HD-associated depression differs from that in the general population. Whilst preclinical studies, clinical data, and treatment responses suggest striking similarities, there are also some apparent differences. We discuss various molecular and cellular mechanisms which may contribute to depression in HD, and whether they may generalize to other depressive disorders. The autosomal dominant nature of HD and the existence of models with excellent construct validity provide a unique opportunity to understand the pathogenesis of depression and associated gene-environment interactions. Thus, understanding the pathogenesis of depression in HD may not only facilitate tailored therapeutic approaches for HD sufferers, but may also translate to the clinical depression which devastates the lives of so many people.

## Introduction

Huntington’s disease (HD) is a progressive neurodegenerative disorder that affects mood, cognition, and movement. HD provides a unique opportunity to study pathogenesis from its earliest stages, as the genetic cause of this autosomal dominant disease, with its array of divergent symptoms, is known. HD is caused by an abnormal expansion mutation of a tract of CAG trinucleotide tandem repeats close to the 5′ end of the *huntingtin* gene on chromosome 4 (The Huntington’s Disease Collaborative Research Group, 1993). HD was first described by the physician George Huntington in 1872, and our understanding of the mechanisms underlying HD has increased exponentially in the 20 years since the tandem repeat expansion in *huntingtin* was found to be the causative gene mutation. Despite this, a cure for this disease remains elusive. In light of this, much attention has been focused on symptom management. As a late onset disease, alleviation of especially early symptoms can greatly lengthen and improve the largely normal and productive periods of patients’ lives. HD presents with a classic triad of relentlessly progressive symptoms. Diagnosis is based on the appearance of motor symptoms, which most commonly appear in patients in the fourth or fifth decade of life. Some rare cases of juvenile onset are seen, with motor symptoms manifesting during or prior to teenage years; however juvenile onset HD, which is caused by exceptionally long CAG repeat expansions, accounts for only about 5% of all HD cases (Nance and Myers, 2001). Completing the triad are cognitive and psychiatric symptoms, which can arise years, often decades, prior to the inception of motor symptoms. There are also a plethora of non-CNS, peripheral symptoms, although it is not yet clear whether specific symptoms originate from cellular dysfunctions in central and/or peripheral tissues (van der Burg et al., 2009).

Psychiatric symptoms abound in HD (Leroi et al., 2002), including psychosis (Lovestone et al., 1996), irritability, anxiety, apathy (van Duijn et al., 2007), and obsessive and compulsive symptoms (Beglinger et al., 2008; van Duijn et al., 2008). However, depression is among the most prevalent of psychiatric symptoms, with a lifetime prevalence of major depression reported to be up to and over 50% in patients; moreover, depression is often diagnosed years if not decades prior motor symptom onset (Shiwach, 1994; Naarding et al., 2001; Paulsen et al., 2001, 2005; Duff et al., 2007; van Duijn et al., 2008; Gargiulo et al., 2009). This occurrence is hugely disproportionate when compared to lifetime prevalence of major depression in the general population, which has been found to be around 15% (Hasin et al., 2005; Kessler et al., 2005).

More than merely prevalent, depression has been rated by patients as one of the most debilitating symptoms of HD, affecting perceived quality of life more so than motor or even cognitive aspects of the disease (Ho et al., 2009). Depression increases the risk of suicide (Jensen et al., 1993; Harris and Barraclough, 1998; Cavanagh et al., 2003). In HD, almost 30% of patients attempt suicide at least once and death due to suicide among HD patients is four times that of the normal populace – a similar rate of death by suicide to that of those suffering from affective disorders (Farrer, 1986; Inskip et al., 1998). The presence of depression also leads to negative collateral effects such as the hastening of cognitive decline (Nehl et al., 2001; Smith et al., 2012). Whilst there is currently no cure available for HD, depression is largely seen as treatable. However, understanding of the etiology and pathophysiology of this early-onset symptom is required to allow early detection of depression and to apply the most suitable treatments.

Huntington’s disease is the most notorious of a group of neurodegenerative diseases caused by an expansion of a CAG trinucleotide repeat encoding poly-glutamine (poly-Q). Other diseases in this group include dentatorubropallidoluysian atrophy (DRPLA), spinocerebellar ataxia type 3 (SCA-3), spinal bulbar muscular atrophy (SBMA), and spinocerebellar ataxia types 1, 2, 6, 7, and 17 (David et al., 1997; Kakizuka, 1997; Koshy and Zoghbi, 1997; Robitaille et al., 1997; Ross, 1997; Nakamura, 2001; Nakamura et al., 2001). SCA patients show depression prevalence of between 15 and 17% in a large study with 526 genetically confirmed and clinically affected patients (117 SCA1, 163 SCA2, 139 SCA3, and 107 SCA6) (Schmitz-Hübsch et al., 2011), a rate similar to the prevalence in the general population (∼15%) and certainly less than that seen in HD. Other members of this group are very rare and studies have not been done specifically examining rates of depression. The overrepresentation of depression in HD may be due to the neurological importance of the huntingtin protein, its ubiquitousness and the subsequent consequences of the mutation.

Whilst the link between the length of CAG expansion and speed of deterioration has been established, evidence from the literature does not support the idea that depression in HD is affected by the length of CAG repeats. In a study with a cohort of 79 HD patients, no correlations were observed between CAG repeat length and personality change, psychosis, depression, or non-specific alterations (Weigell-Weber et al., 1996). No relationship was discerned even after correcting for predicted age of neurological onset in neurologically asymptomatic patients (Berrios et al., 2001). A more recent study examining 72 HD patients found that whilst the number of CAG repeats associated negatively with the age of onset of psychiatric disorders, neither the probability of developing psychiatric disorders nor the severity of psychiatric symptoms was correlated with the number of CAG repeats (Vassos et al., 2008). It therefore seems that although higher repeat number is associated with faster disease progression as a whole, it is insufficient to induce depression.

Although the ultimate cause of depression in HD may be different from that of depression in the HD gene-negative population (which we will refer to forthwith as “clinical depression”), recent research has shown that there are certainly perceptible similarities in the symptoms and even physiological anomalies. These similarities are worth examining as they may hold clues to the etiology of depression in HD and may be informative in establishing how best to treat this debilitating affective disorder. Various aspects of HD pathogenesis, including disrupted transcription, trafficking, signaling, homeostasis, synaptic and neuronal function, have been recently reviewed (Milnerwood and Raymond, 2010; Raymond et al., 2011; Ross and Tabrizi, 2011; Nithianantharajah and Hannan, 2012). Here, we discuss potential molecular and cellular mechanisms specifically involved in the etiology of depression in HD. This includes evidence implicating the dysregulation of serotonergic signaling, alteration of hypothalamic-pituitary-adrenal (HPA)-axis activity, and disruption of BDNF expression and trafficking as key pathogenic processes.

## Diagnosing Depression in HD

Notwithstanding evidence of overwhelming prevalence of psychiatric symptoms, treatment of psychopathologies in HD has been scantly examined. Studies of the efficacy of various drugs in treating these symptoms have been few and far between, mostly with small cohorts, often case reports of a single patient with no controls (Naarding et al., 2001). While conventional antidepressants have been applied in the treatment of depression in HD, the effectiveness of different types of antidepressants, doses, treatment durations, and other factors have not been thoroughly examined. Having a different origin of disease, i.e., the mutation in the *huntingtin* gene, it is possible that disparities exist in HD which may translate to more efficacious treatment strategies. For instance, a study has found that passivity is a risk factor to earlier disease onset versus patients who were more active (Trembath et al., 2010). This reflects findings in mouse models where transgenic mice raised in enriched cages which provided enhanced sensory stimulations were rescued from depressive-like behavioral phenotypes seen in mice raised in standard cages (Pang et al., 2009; Du et al., 2012; Renoir et al., 2013). This suggests enhanced engagement and participation may have benefits in the treatment of depression. Little has been done with regards to behavioral therapy in HD but a study has shown that remotivational therapy improving quality of life of HD patients (Sullivan et al., 2001). A recent study on the use of antidepressants in pre-onset HD gene carriers found that about 22% of 787 prodromal patients are already on antidepressants, much more than in the control group, with around 13% on antidepressants (Rowe et al., 2012). However, a consensus on the prevalence of depression in HD has yet to be reached. The inconsistency in rates of depression reported between different studies is due to a variety of factors such as small sample sizes, differences in methodologies utilized, lack of control groups, and differences in the disease stages of participants (van Duijn et al., 2007). In particular, the usage of different assessment tools [Unified Huntington’s Disease Rating Scale (UHDR), Diagnostics and Statistics Manual of Mental Disorders (DSM), Neuropsychiatric Inventory (NPI), Beck Depression Inventory-II (BDI-II), Hamilton Rating Scale for Depression (Ham-D), Hospital Anxiety and Depression Scale (HADS), Depression Intensity Scale Circles (DISCs)] have resulted in very disparate estimations of the prevalence of depression in HD, ranging from 33 to 69%.

This large variation highlights the endogenous differences between how depression manifests with and without the HD gene mutation, causing the use of tools not tailored for depression in HD to produce inconsistent results. For example, standard rating scales for clinical depression contain items relating to symptoms of HD that are present regardless of the presence or absence of depression and therefore may skew the diagnosis. Weight loss and sleep disturbances are items included in many diagnostic tools as symptomatic of depression even though they are common symptoms in HD patients without depression. Therefore these symptoms may well be nothing but red herrings if included for diagnosis of depression in HD. Scales that contain less “somatic” items, such as the HADS and the DISCs were better able to accurately identify depression in HD (De Souza et al., 2010). Comparison between the BDI-II and the Ham-D found that the items best able to discriminate depression in HD patients, other than depressed mood, which had high correlations [correlation coefficient (*r*) = 0.834 for the BDI and 0.917 for the Ham-D], are those items measuring thoughts and attitudes around mood such as “discouraged about the future” (*r* = 0.653) and “satisfaction in life” (*r* = 0.64). Weight loss (*r* = 0.193 for BDI and 0.128 for Ham-D), loss of appetite (*r* = 0.319 for BDI, 0.297 for Ham-D), and other vegetative symptoms associated with clinical depression were, on the other hand, poor indicators of depression in HD (Rickards et al., 2011). Depression is notoriously heterogeneous in its presentation and these findings suggest possible dominance of a different cluster of symptoms in HD compared to most clinical depression patients. Accurate discriminators of depression in HD are required for better screening, diagnosis, and the gaging of treatment efficacy; areas that have not so far been systematically studied. Whether or not the differences reflected in the screening tools point to underlying pathophysiological divergence between depression in HD and clinical depression require further investigations. Besides the obvious consequences of depression itself, another important reason for the accurate and early detection of depression in HD is that depression has the negative effects of hastening cognitive decline and correlates with poorer cognitive performance in prodromal patients (Nehl et al., 2001; Smith et al., 2012), suggesting that earlier intervention can prolong the functional years of a patient’s life.

It is tempting to speculate, and indeed has been a predominant view, that depression in HD is a natural consequence to the reactive stress induced by the knowledge of being at risk of inheriting the disease and/or of positive genetic or clinical diagnosis of HD (Shiwach and Norbury, 1994). Whilst psychosocial stress undoubtedly adds to the psychological burden inducing depression (Codori et al., 2004; Larsson et al., 2006), it alone is now regarded as insufficient to fully account for the psychiatric co-morbidity. Prior to clinical onset, HD gene-positive carriers presented a higher current prevalence of major depression despite being ignorant of their gene status at the time of psychiatric assessment (Horowitz et al., 2001; Julien et al., 2007). That overrepresentation suggests that depression in HD patients is a behavioral manifestation of early neuropathology. Interestingly, the greater susceptibility of females to developing depression in the general population is also apparent within the HD population (Zielonka et al., 2012). These clinical aspects have been recapitulated by studies of the R6/1 transgenic and knock-in HdhQ111 mouse models (that are by definition unaware of their gene status) which report a female-specific depression-like behavioral phenotype (Pang et al., 2009; Pouladi et al., 2009; Du et al., 2012; Orvoen et al., 2012; Renoir et al., 2012), circumventing the caveat of psychosocial stress. Whilst sexual dimorphism has been reported in the CAG(n51) transgenic HD rats, the nature of the affective dysfunction in this model is less clear (Bode et al., 2008; Faure et al., 2011). Depressive-like behaviors have also been identified in the YAC transgenic HD mice (Pouladi et al., 2009), suggesting that it is a fundamental aspect of HD phenotypes. The study of other rodent models of HD might be relevant for the understanding of other specific psychiatric aspects of HD such as anxiety (Orvoen et al., 2012; Abada et al., 2013).

Although the pathophysiology leading to depression in HD arises from a different source than those of clinical depression, the successes of antidepressant treatments in ameliorating depression in HD, albeit mostly from case studies, suggest similarities in the underlying pathophysiology. Therefore it is important to take advantage of the extensive knowledge garnered in the study of clinical depression and to compare the two diseases in order to apply the most effective and tailored treatment for depression in HD.

## The Serotonergic System

Symptomatic treatment of depression in HD and the efficacy of the range of antidepressant drugs often prescribed to HD patients have not been thoroughly examined. Case studies report benefits of selective serotonin reuptake inhibitors (SSRIs), selective noradrenergic reuptake inhibitors (SNRIs) (venlafaxine), atypical antipsychotics (olanzepine), monoamine oxidase inhibitors (MAOI), tetracyclic, and tricyclic antidepressants on small numbers of patients (Patel et al., 1996; Squitieri et al., 2001; Bonelli et al., 2003; Ciammola et al., 2009). A study of 26 HD patients with diagnoses of major depression treated with venlafaxine for 4 weeks showed significant improvement albeit a high rate of side effects such as irritability (Holl et al., 2010). In a more recent study by Rowe et al. (2012) of 787 prodromal HD participants, it was reported that 20% of prodromal patients were prescribed antidepressants with the vast majority using SSRIs (e.g., paroxetine, fluoxetine, and sertraline). However, the effectiveness SSRIs versus other types of antidepressants in diminishing the depression symptoms has yet to be specifically documented. Indeed the overall effectiveness of SSRI interventions in treating depression in HD has not been studied despite its frequent use.

Dysregulation of the serotonin (5-HT) signaling system has long been scrutinized in the field of clinical depression as an etiological cause (Castro et al., 1998; Yohrling et al., 2002). Compromising the 5-HT system affects its downstream elements; 5-HT upregulates the expression of cyclic adenosine monophosphate (cAMP) (Vaidya and Duman, 2001), which ultimately results in the activation of cAMP response element-binding protein (CREB). A loss of CREB signaling impairs adult hippocampal neurogenesis and disrupts normal hippocampal function (Jacobs et al., 2000; Urani et al., 2005), both of which have been proposed to be key pathologies of depression (Petersen et al., 2008; Lucassen et al., 2010). Based on findings from animal models, CREB signaling in HD is also disrupted, due to sequestration of its binding partner, CREB binding protein (CBP). Thus, this is a common molecular pathology that the disease shares with depression, and one which, along with the target gene BDNF as described below, is likely to mediate the deficits in hippocampal neurogenesis which is the cellular consistently observed in animal models of HD. Chronic treatment of different mouse models of HD with a variety of SSRIs have been found to rescue the neurogenesis deficits but there has yet to be a study demonstrating a possible rescue of neurogenesis through modulation of CREB signaling. That would be an interesting avenue of investigation for the future since compounds that increase phosphorylation of CREB and cAMP levels are reportedly associated with a rescue of cognitive deficits in the R6/1 transgenic mouse model (Giralt et al., 2013).

Serotonin reuptake inhibitors work in part by desensitizing 5-HT1A autoreceptors (Dawson et al., 2002; Rossi et al., 2008), which act to inhibit 5-HT production. Desensitization of these autoreceptors therefore increases 5-HT neurotransmission (Blier et al., 1998). It was also found that a single nucleotide polymorphism (SNP) on the promoter of 5-HT1A receptor which leads to the over-expression of 5-HT1A autoreceptors resulted in increased susceptibility to developing major depression in those with the mutation (Albert and Francois, 2010). As levels of 5-HT in the human brain can only be measured post-mortem, markers are used in patients to reflect 5-HT levels. One of these is measuring the primary metabolite of 5-HT, 5-hydroxyindoleacetic acid (5-HIAA) in the cerebrospinal fluid (CSF), which is deemed to reflect brain 5-HIAA levels (Wester et al., 1990). It has been found that 5-HIAA is reduced in the CSF of a sizeable proportion of patients with major depression, a finding replicated by *post-mortem* studies (Asberg, 1976; Asberg et al., 1976; van Praag and Plutchik, 1984; Roy et al., 1989). Reduced 5-HIAA levels have also been reported in HD patients, which is a clear indicator that there is a dysregulation of serotonin metabolism in HD (Caraceni et al., 1977; Jongen et al., 1980). Closely related, monoamine oxidase A, an enzyme that metabolizes 5-HT thereby decreasing the amount of available 5-HT, was elevated significantly in many brain regions of depressed patients (Meyer et al., 2006). This abnormal increase in metabolism of 5-HT has similarly been documented in the putamen and substantia nigra pars compacta of the basal ganglia, and in the pons of HD brains through quantitative enzyme radioautography (Richards et al., 2011). Interestingly, platelet monoamine oxidase activity was reported to be unchanged in HD patients with overt symptoms (Markianos et al., 2004) which suggests a dissociation between central and peripheral pathology in HD. Tryptophan, an essential amino acid from which serotonin is synthesized, has also been found to be reduced in a large proportion of patients with depression (Moller et al., 1983; Quintana, 1992). Experimentally induced tryptophan depletion via a special diet resulted in rapid relapse in remitted depression patients hours after ingestion of diet (Delgado et al., 1990). Plasma total and protein-bound tryptophan levels were found to be reduced in HD patients (Belendiuk et al., 1980) and while oral tryptophan administration increases blood 5-HT levels in healthy individuals, this response not observed in HD patients which is a further demonstration of a disease-associated impairment of 5-HT metabolism (Christofides et al., 2006). A recent study looking at the raphe nucleus, where 5-HT synthesis occurs, found a significant correlation between the level of depression in HD patients and stem raphe echogenicity using transcranial sonography (Krogias et al., 2011). This is similar to what is seen in clinical depression patients (Walter et al., 2007), further strengthening the evidence that an abnormality in 5-HT production may underlie both diseases. Recent work on compounds which influence tryptophan metabolism rescue neurodegeneration in the R6/2 mouse model of HD (Zwilling et al., 2011) but the effectiveness of such compounds to treat depressive behaviors have yet to be determined.

Besides changes to 5-HT levels, dysregulation of the receptors of 5-HT has also been implicated in depression. Major depression has been implicated with increased 5-HT1A autoreceptor density in the dorsal raphe nucleus (Stockmeier et al., 1998). Reduced post-synaptic 5-HT1A/2 receptor function has also been linked with depression with patients who had suffered major depression exhibiting global reduction in 5-HT1A and 5-HT2A receptor binding (Drevets et al., 1999, 2007; Messa et al., 2003; Moses-Kolko et al., 2008). This is reflected in animal models where genetic 5-HT1A receptor knock-out mice showed phenotypes of anxiety (Heisler et al., 1998; Parks et al., 1998; Ramboz et al., 1998) whereas over-expression of 5-HT1A receptor reduced anxiety-related behaviors (Kusserow et al., 2004). In *post-mortem* HD brains, reduced 5-HT1 receptor binding has been reported in the putamen, hippocampus (Cross et al., 1986), and in the basal ganglia and substantia nigra (Waeber and Palacios, 1989). Similar reductions in 5-HT1A receptor binding have been reported in the R6/2 transgenic mouse model, as well as a marked decrease in enzymatic activity of tryptophan hydroxylase (TPH) activity (the rate limiting enzyme for the biosynthesis of 5-HT) (Yohrling et al., 2002). The decrease in receptor binding is likely attributable to down-regulation of mRNA levels of various 5-HT receptors which is evident in R6/1 HD mice (Pang et al., 2009). All these culminate in abnormal physiological responses to the administration of 5-HT1A receptor agonist 8-OH-DPAT by R6/1 HD mice (Renoir et al., 2011a, 2012), thereby demonstrating how molecular neuropathology manifests as disease pathophysiology. Interestingly, chronic application of the SSRI sertraline was able to correct the hypersensitivity of the 5-HT1A autoreceptor in female R6/1 mice as well as depression-like phenotype (Renoir et al., 2012), suggesting an etiological link between disturbances to the 5-HT system with the HD-associated depression-like phenotype.

## The HPA-Axis and Stress Responses

The HPA-axis is the major endocrine system responsible for stress adaptation. Stress activates the axis, resulting in the production of the stress hormone cortisol (corticosterone in rodents) (Papadimitriou and Priftis, 2009). Abnormally elevated HPA-axis activity is one of the most replicated biological findings in major depression (Pariante and Lightman, 2008; Stetler and Miller, 2011). Cortisol levels are usually tightly regulated because prolonged exposure to cortisol is damaging to the brain: reducing neurogenesis (Cameron and Gould, 1994; Wong and Herbert, 2006; Brummelte and Galea, 2010) and increasing apoptosis in the hippocampus (Sapolsky, 1986; Crochemore et al., 2005; Andrés et al., 2006; Liu et al., 2011) resulting in atrophy in the hippocampus of rats (Woolley et al., 1990) and monkeys (Sapolsky et al., 1990) as well as neuronal atrophy (Cerqueira et al., 2005a) and volume reduction in the prefrontal cortex (Cerqueira et al., 2005b). Dysregulation of the 5-HT neurotransmission has been linked with the HPA-axis abnormalities (Hery et al., 2000; Froger et al., 2004). The serotonin system is able to modulate anxiety and depression (Meaney et al., 1994; Ramboz et al., 1998; Harada et al., 2008; Brummett et al., 2012), possibly by regulating the HPA-axis through influencing glucocorticoid receptor (GR), whose activation is important in regulating the 5-HT system both *in vivo* and *in vitro* (Lanfumey et al., 2000; Erdeljan et al., 2001; Wang et al., 2009; Falkenberg and Rajeevan, 2010; Belay et al., 2011). For example, it has been found that serotonin neurotransmission is modulated by HPA-axis activity, with GR activation increasing 5-HT1A signaling (Hesen and Joels, 1996). Adrenalectomy reduced dentate gyrus neuronal morphology and this effect was reversed by using the 5-HT1A agonist ipsapirone (Huang et al., 1997). But sustained elevation of corticosteroid significantly reduced 5-HT1A receptor mRNA level as well as 5-HT1A binding density in the hippocampus and this is reversed by chronic administration of antidepressants (Lopez et al., 1998).

Studies have also described HPA-axis hyperactivity in HD patients. Earlier studies with small sample sizes found higher basal cortisol levels in moderate stage HD patients compared to controls at both morning (Leblhuber et al., 1995) and evening (Heuser et al., 1991). More recently, hyperactivity of the HPA-axis has been found in a small sample of clinically diagnosed, early stage HD patients compared to controls, with higher 24 h cortisol production, particularly in the morning and early afternoon periods (Aziz et al., 2009). Examining salivary cortisol in pre-symptomatic HD patients in comparison to controls, subtle but altered cortisol awakening responses were found, with higher cortisol concentration in patients at early morning periods, just after awakening (van Duijn et al., 2010). This indicates that HPA-axis dysregulation is an early pathophysiology in HD. However, another study with a comparatively large sample of patients found increased urine cortisol levels but only in moderate to late clinically diagnosed patients whereas pre-symptomatic and early stage patients did not display any differences in urine cortisol compared to 68 healthy controls (Björkqvist et al., 2006). But it is worthy to note that in this study, samples were collected in a narrow time window (14:00–17:00), when cortisol level is ebbing, leaving the possibility that the difference identified in the previous studies was masked in the early stage patients examined. Furthermore, analysis of patients was confounded by limitations to recruitment such as imbalance of gender (or indeed disregarding gender as a factor), differences in stages of disease progression as well as environmental confounders such as smoking, alcoholism, depression, and use of psychotropic medications.

Increased baseline corticosterone was initially found in R6/2 mice from an early age (Björkqvist et al., 2006). However, because of their rapid motor onset and deterioration, the R6/2 model is not an optimal one for examining pre-motor onset symptoms and pathophysiologies. Recently, it was found that at a pre-motor onset age, female R6/1 mice exhibit increased corticosterone release after physiological and pharmacological stresses whilst baseline levels did not differ between WT and R6/1 females (Du et al., 2012). This is more akin to what is seen in early, pre-motor symptomatic HD patients with little change in baseline cortisol levels, suggesting that in prodromal patients, differences may only become apparent when the HPA-axis is induced by stressors. Interestingly, the sex-dimorphic display of this physiological abnormality correlates with prior findings of depression-like behavioral phenotypes in pre-motor onset female R6/1 mice that were also absent in the males of the same age (Pang et al., 2009; Renoir et al., 2011b). Curiously, using pharmacological means as well as *in vitro* analysis, it was found that the source of the hyperactivity in the female R6/1 mice is the adrenal gland (Du et al., 2012). Studies examining the adrenal glands in HD have so far been scant. However, this finding raises the possibility of an early peripheral change which may influence central brain function. More research is required to examine the HPA-axis of HD patients in terms of its regulation and response to stress. Whilst adrenal hyperplasia has been found in victims of suicide (Szigethy et al., 1994) and a few studies reporting increased adrenal volume in depressed patients compared to controls, the lack of numbers and heterogeneity between studies makes it hard to draw useful conclusions about the role adrenal-specific pathology may play in the development of depression (Kessing et al., 2011).

## BDNF and Neurotrophin Signaling

Depression is a complex affective disorder and is thought to result at least partially from an inability of the brain to make appropriate adaptations to environmental stressors, and may involve impaired neural plasticity (Duman et al., 2000; Duman, 2002; Manji et al., 2003; Czeh and Simon, 2005). This is evidenced by studies showing altered brain structures in depression subjects such as reduced cell number, cell density, and body size as well as reduced glial density in frontal cortical and hippocampal brain regions (Ongur et al., 1998; Cotter et al., 2001; Beasley et al., 2002; Rajkowska, 2002). These observations led to the hypothesis that a loss of neurotrophic factors, which are vital for the survival, development, and maintenance of neurons (Lewin and Barde, 1996; McAllister, 2001) as well as regulating synaptic and morphological plasticity (McAllister et al., 1999; Thoenen, 2000), is directly involved in the pathophysiology of depression and that its restitution may lie at the heart of successful treatment (Altar, 1999; Duman, 2004). Indeed, recently it was found that HD patients exhibited a significant decrease in peripheral BDNF gene expression (Krzyszton-Russjan et al., 2012). HD patients (Ciammola et al., 2007) and rodent models of HD (Strand et al., 2007) also show reduced levels of BDNF protein. Huntingtin has important roles in regulating both the transcription (Zuccato et al., 2001) and trafficking (DiFiglia et al., 1995; Gauthier et al., 2004) of BDNF, actions that are affected by the mutation in HD.

BDNF helps support the survival and integrity of existing neurons and encourage growth, migration, and differentiation of new neurons and synapses in the central as well as peripheral nervous systems (Cowansage et al., 2010). It also enhances neurogenesis in the hippocampus (Zigova et al., 1998; Benraiss et al., 2001; Pencea et al., 2001). The expression of BDNF is regulated via neuronal activity through calcium mediated mechanisms (Tabuchi, 2008) whilst its receptor tyrosine kinase-coupled receptor (TrkB), is also regulated in an activity-dependent manner (Nagappan and Lu, 2005). As compromised synaptic and structural plasticity is significantly associated with depression, the importance of BDNF in depression has therefore garnered considerable attention. Lower levels of BDNF in depression patients suggest a role of BDNF in the pathogenesis of depression (Yoshimura et al., 2010; Yoshida et al., 2012). Serum BDNF was found to be significantly reduced in antidepressant-naïve depression patients compared to those who were treated with antidepressants and there was a significant negative correlation between BDNF levels and the Hamilton Rating Scale for Depression (Shimizu et al., 2003). Hippocampal BDNF levels in post-mortem brains of depression patients were found to be higher in those treated with antidepressants at time of death (Chen et al., 2001; Karege et al., 2005). This suggests that antidepressants increase BDNF levels and may account, at least in part, for their potency. In animal models, a similar result was seen. It was found that early life stress lowers BDNF and alters stress sensitivity later in life (Cirulli et al., 2009), whereas increases in BDNF, either via social enrichment or direct infusion into the brain, was found to reduce susceptibility to depression-like behavior (Siuciak et al., 1997; Cirulli et al., 2010).

However, studies also found that increases in BDNF through communal nesting induced an anxiety phenotype by reducing latency to immobility and increasing immobility time in the forced-swim test (FST) (Branchi et al., 2006). Other studies examining the role of BDNF in depression have also found that heterozygous knock-out of BDNF did not alter anxiety profile on the elevated-plus maze (Montkowski and Holsboer, 1997; MacQueen et al., 2001; Gorski et al., 2003). BDNF heterozygotes or mice expressing the dominant negative TrkB receptor TrkB.T1, did not show any differences from WT mice in their performance on the FST, therefore showing that reduced BDNF signaling does not cause depression *per se* (Saarelainen et al., 2003). However, the fact that a lack of BDNF signaling negated the effects of antidepressant treatment argues that whilst it is not predictive of depression, it is integral for the potency of antidepressant action.

BDNF seems to mediate the positive effect of several classes of antidepressants including SSRIs, tricyclics, SNRI, and MAOI, which have been found to increase BDNF in serum, cortical astrocytes, prefrontal cortex, and hippocampus (Duman and Monteggia, 2006; Allaman et al., 2011; Molendijk et al., 2011). It is also upregulated by exercise and environmental enrichment, contributing to beneficial effects such as induction of cell survival, proliferation, and dendritic development, leading to augmented cognitive outcomes (Acheson et al., 1995; Cotman and Berchtold, 2002; Choi et al., 2009; Sun et al., 2010; Kazlauckas et al., 2011). Environmental enrichment and exercise (voluntary wheel running) have been found to rescue BDNF deficits in R6/1 HD mice as well as ameliorating affective, cognitive, and motor abnormalities (van Dellen et al., 2000; Spires et al., 2004; Pang et al., 2006, 2009; Nithianantharajah et al., 2008; Zajac et al., 2010; Renoir et al., 2013). A diet rich in omega-3 fatty acids, which has been shown to have positive effects on depression (Freeman, 2009), also increases BDNF levels in the hippocampus (Venna et al., 2009).

In BDNF-deficient mice, the behavioral effects of antidepressants were abolished (Saarelainen et al., 2003; Monteggia et al., 2004). TrkB T1 over-expressing transgenic mice, which show reduced TrkB activation in the brain, are resistant to the effects of antidepressants (Saarelainen et al., 2003), whereas over-expression of TrkB led to resistance to depression-like behavior, with SSRI administration unable to further increase this resistance (Koponen et al., 2005) indicating that TrkB signaling is required for the behavioral benefits of antidepressants. These findings suggest that BDNF may be a key molecule involved in various antidepressant treatment strategies. A possible mechanism mediating the antidepressant-induced increase of BDNF is the upregulated expression of CREB, a transcription factor that upregulates BDNF and TrkB (Nibuya et al., 1996). Interestingly, CREB-mediated transcription regulation requires the aid of CBP, whose expression is downregulated by mutant huntingtin (Kazantsev et al., 1999; Nucifora et al., 2001).

Antidepressants are widely used in the treatment of HD patients (Sackley et al., 2011). Recent studies suggest that chronic treatment with the SSRIs fluoxetine or sertraline increased hippocampal neurogenesis, ameliorated cognitive deficits, and depression-like behavioral symptoms in R6/1 mice (Grote et al., 2005; Renoir et al., 2012) and increased BDNF levels and neurogenesis in R6/2 mice (Peng et al., 2008). Chronic antidepressant treatment in depressed patients resulted in upregulation of CREB protein expression (Nibuya et al., 1996), CREB phosphorylation (Saarelainen et al., 2003), BDNF (Chen et al., 2001), and TrkB (Bayer et al., 2000) in the hippocampus. BDNF has been proposed to be a mediator of the effects of antidepressants (Koponen et al., 2005), by augmenting the survival and differentiation of adult-born neurons in the dentate gyrus (Groves, 2007). These results led to the hypothesis that depression in HD coincides with decreased activity in the serotonin-CREB-BDNF-TrkB pathway, resulting in cellular dysfunction and reduced neurogenesis in the hippocampus.

**Figure 1:**
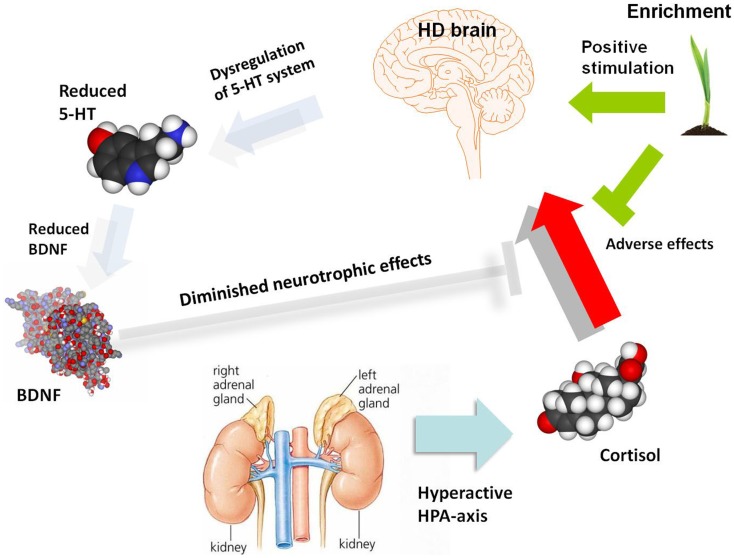
**A schematic diagram illustrating potential molecular and cellular mediators of HD pathogenesis, with a focus on serotonergic and neurotrophin signaling as well as the HPA-dysfunction which may give rise to depression and associated symptoms**. The role of environmental enrichment in ameliorating specific aspects of molecular, cellular, and systems dysfunction is also illustrated. There are clearly many other molecular and cellular candidates implicated in HD pathogenesis which are not shown in this diagram, which outlines a specific hypothesis regarding the etiology of depression in HD. 5-HT, serotonin; BDNF, brain-derived neurotrophic factor; HD, Huntington’s disease; HPA-axis, hypothalamic-pituitary-adrenal axis.

The link between HPA-axis, depression, and BDNF has been explored in rodent models of depression and given much attention. Social stress has been widely used as a useful model of depression (Henn and Vollmayr, 2005). Stressors such as forced immobilization (Smith et al., 1995) and social defeat (Pizarro et al., 2004) were found to decrease BDNF expression in the hippocampus and cortical and subcortical regions of rodent models. Induced elevation of corticosterone, mimicking the effect of stress, has also been associated with reduced levels of BDNF mRNA and protein in the hippocampus and frontal cortex of rodent models (Schaaf et al., 1997, 1998; Chao et al., 1998; Dwivedi et al., 2006). Adrenalectomy surgery caused an increase of BDNF in the hippocampus (Chao et al., 1998), whilst chronic GR activation reduces both CREB phosphorylation and BDNF expression (Focking et al., 2003). This suggests regulatory ability of glucocorticoids on BDNF expression. GR was also found to interact with the BDNF receptor TrkB and corticosterone reduces TrkB-GR interaction, causing reduced BDNF-triggered glutamate release and BDNF-stimulated PLC-γ (Numakawa et al., 2009). Thus, taken together, increased HPA-axis activity may initiate a chain reaction, leading to altered 5-HT signaling, reduced CREB-mediated transcription of BDNF and damage to the hippocampus and other brain regions, which in turn, reduces negative feedback on the HPA-axis in a negative cycle (Figure 1). The molecular and cellular processes may be impacted by environmental modulators, such as the cognitive stimulation and physical exercise induced by environmental enrichment. Complex gene–gene interactions and associated gene-environment interactions are presumably responsible for the variable incidence of depression both within HD patients (where each tandem repeat expansion mutation is embedded in a genome possessing a range of genetic modifiers) and the general population. Elucidation of this complexity at molecular, cellular, and systems levels will require a new generation of sophisticated animal models and clinical investigations.

## Summary

As a cure for HD remains elusive, effective strategies targeting symptom management are of great interest. This is especially pertinent with early onset psychiatric and cognitive symptoms, which tend to occur in the most productive years. Depression, being both early onset and having devastating consequences, is a priority target.

Collectively, HD shares certain hallmarks with a large portion of clinical depression patients: namely, disturbances of the 5-HT system, BDNF expression, and HPA-axis dysregulation. This is not perhaps surprising as the use of conventional antidepressants have shown to be equally efficacious in treating depression in HD. However, due to the relentless deterioration of the brain seen in HD, it is paramount to diagnose depression both early and accurately in the hope of stemming the decline with appropriate interventions. The inconsistent diagnostic results reflect the need for a tailored diagnostic tool for HD, taking into account many somatic symptoms such as weight loss which often seem to have little to do with depression. Furthermore, besides antidepressants such as SSRIs, other methods may be examined in detail as potential therapeutics. Strategies such as exercise interventions, occupational therapy, and other cognitive/behavioral treatments are under-utilized in HD and worthy of systematic investigation, both on their own and as adjuncts to pharmacological treatments.

Ultimately, the treatment of HD may require a sophisticated understanding of the pathogenesis of psychiatric, cognitive and motor symptoms, and the source of their heterogeneity in clinical populations. For example, it would be expected that genetic polymorphisms/mutations which predispose to depression in the general population might have additive effects on predisposition when combined with the HD mutation. Similarly, the kind of stressors which can trigger depression in genetically vulnerable individuals might be equally toxic for those who are HD gene-positive. A natural consequence of such detailed insights into pathogenesis could be pharmacogenomics, polypharmacy, and other tailored therapeutic approaches to improve the lives of those suffering from HD and depression.

## Conflict of Interest Statement

The authors declare that the research was conducted in the absence of any commercial or financial relationships that could be construed as a potential conflict of interest.

## References

[B1] Abada Y. S. Schreiber R. Ellenbroek B. (2013). Motor, emotional and cognitive deficits in adult BACHD mice: a model for Huntington’s disease. Behav. Brain Res. 238, 243–251 10.1016/j.bbr.2012.10.039 23123142

[B2] Acheson A. Conover J. C. Fandl J. P. DeChiara T. M. Russell M. Thadani A. (1995). A BDNF autocrine loop in adult sensory neurons prevents cell death. Nature 374, 450–453 10.1038/374450a0 7700353

[B3] Albert P. R. Francois B. L. (2010). Modifying 5-HT1A receptor gene expression as a new target for antidepressant therapy. Front. Neurosci. 4:35 10.3389/fnins.2010.00035 20661455 PMC2907233

[B4] Allaman I. Fiumelli H. Magistretti P. J. Martin J. L. (2011). Fluoxetine regulates the expression of neurotrophic/growth factors and glucose metabolism in astrocytes. Psychopharmacology (Berl.) 216, 75–84 10.1007/s00213-011-2190-y 21301813

[B5] Altar C. A. (1999). Neurotrophins and depression. Trends Pharmacol. Sci. 20, 59–61 10.1016/S0165-6147(99)01309-7 10101965

[B6] Andrés S. Cárdenas S. Parra C. Bravo J. Greiner M. Rojas P. (2006). Effects of long-term adrenalectomy on apoptosis and neuroprotection in the rat hippocampus. Endocrine 29, 299–307 10.1385/ENDO:29:2:299 16785605

[B7] Asberg M. (1976). Treatment of depression with tricyclic drugs – pharmacokinetic and pharmacodynamic aspects. Pharmakopsychiatr. Neuropsychopharmakol. 9, 18–26 10.1055/s-0028-1094473 10583

[B8] Asberg M. Traskman L. Thoren P. (1976). 5-HIAA in the cerebrospinal fluid. A biochemical suicide predictor? Arch. Gen. Psychiatry 33, 1193–1197 10.1001/archpsyc.1976.01770100055005 971028

[B9] Aziz N. A. Pijl H. Frolich M. van der Graaf A. W. Roelfsema F. Roos R. A. (2009). Increased hypothalamic-pituitary-adrenal axis activity in Huntington’s disease. J. Clin. Endocrinol. Metab. 94, 1223–1228 10.1210/jc.2008-2543 19174491

[B10] Bayer T. A. Schramm M. Feldmann N. Knable M. B. Falkai P. (2000). Antidepressant drug exposure is associated with mRNA levels of tyrosine receptor kinase B in major depressive disorder. Prog. Neuropsychopharmacol. Biol. Psychiatry 24, 881–888 10.1016/S0278-5846(00)00115-9 11041531

[B11] Beasley C. L. Cotter D. R. Everall I. P. (2002). Density and distribution of white matter neurons in schizophrenia, bipolar disorder and major depressive disorder: no evidence for abnormalities of neuronal migration. Mol. Psychiatry 7, 564–570 10.1038/sj.mp.4001038 12140779

[B12] Beglinger L. J. Paulsen J. S. Watson D. B. Wang C. Duff K. Langbehn D. R. (2008). Obsessive and compulsive symptoms in prediagnosed Huntington’s disease. J. Clin. Psychiatry 69, 1758–1765 10.4088/JCP.v69n1111 19012814 PMC3658314

[B13] Belay H. Burton C. L. Lovic V. Meaney M. J. Sokolowski M. Fleming A. S. (2011). Early adversity and serotonin transporter genotype interact with hippocampal glucocorticoid receptor mRNA expression, corticosterone, and behavior in adult male rats. Behav. Neurosci. 125, 150–160 10.1037/a0022891 21463019

[B14] Belendiuk K. Belendiuk G. W. Freedman D. X. (1980). Blood monoamine metabolism in Huntington’s disease. Arch. Gen. Psychiatry 37, 325–332 10.1001/archpsyc.1980.01780160095011 6102459

[B15] Benraiss A. Chmielnicki E. Lerner K. Roh D. Goldman S. A. (2001). Adenoviral brain-derived neurotrophic factor induces both neostriatal and olfactory neuronal recruitment from endogenous progenitor cells in the adult forebrain. J. Neurosci. 21, 6718–6731 11517261 10.1523/JNEUROSCI.21-17-06718.2001 PMC6763117

[B16] Berrios G. E. Wagle A. C. Marková I. S. Wagle S. A. Ho L. W. Rubinsztein D. C. (2001). Psychiatric symptoms and CAG repeats in neurologically asymptomatic Huntington’s disease gene carriers. Psychiatry Res. 102, 217–225 10.1016/S0165-1781(01)00257-8 11440772

[B17] Björkqvist M. Petersén A. Bacos K. Isaacs J. Norlén P. Gil J. (2006). Progressive alterations in the hypothalamic-pituitary-adrenal axis in the R6/2 transgenic mouse model of Huntington’s disease. Hum. Mol. Genet. 15, 1713–1721 10.1093/hmg/ddl094 16613897

[B18] Blier P. Pineyro G. el Mansari M. Bergeron R. de Montigny C. (1998). Role of somatodendritic 5-HT autoreceptors in modulating 5-HT neurotransmission. Ann. N. Y. Acad. Sci. 861, 204–216 10.1111/j.1749-6632.1998.tb10192.x 9928258

[B19] Bode F. J. Stephan M. Suhling H. Pabst R. Straub R. H. Raber K. A. (2008). Sex differences in a transgenic rat model of Huntington’s disease: decreased 17beta-estradiol levels correlate with reduced numbers of DARPP32+ neurons in males. Hum. Mol. Genet. 17, 2595–2609 10.1093/hmg/ddn159 18502785

[B20] Bonelli R. M. Mayr B. M. Niederwieser G. Reisecker F. Kapfhammer H. P. (2003). Ziprasidone in Huntington’s disease: the first case reports. J. Psychopharmacol. (Oxford) 17, 459–460 10.1177/0269881103174009 14870962

[B21] Branchi I. D’Andrea I. Sietzema J. Fiore M. Di Fausto V. Aloe L. (2006). Early social enrichment augments adult hippocampal BDNF levels and survival of BrdU-positive cells while increasing anxiety- and “depression”-like behavior. J. Neurosci. Res. 83, 965–973 10.1002/jnr.20789 16477619

[B22] Brummelte S. Galea L. A. (2010). Chronic high corticosterone reduces neurogenesis in the dentate gyrus of adult male and female rats. Neuroscience 168, 680–690 10.1016/j.neuroscience.2010.04.023 20406669

[B23] Brummett B. H. Kuhn C. M. Boyle S. H. Babyak M. A. Siegler I. C. Williams R. B. (2012). Cortisol responses to emotional stress in men: association with a functional polymorphism in the 5HTR2C gene. Biol. Psychol. 89, 94–98 10.1016/j.biopsycho.2011.09.013 21967853 PMC3245751

[B24] Cameron H. A. Gould E. (1994). Adult neurogenesis is regulated by adrenal steroids in the dentate gyrus. Neuroscience 61, 203–209 10.1016/0306-4522(94)90224-0 7969902

[B25] Caraceni T. Calderini G. Consolazione A. Riva E. Algeri S. Girotti F. (1977). Biochemical aspects of Huntington’s chorea. J. Neurol. Neurosurg. Psychiatr. 40, 581–587 10.1136/jnnp.40.6.581 143508 PMC492765

[B26] Castro M. E. Pascual J. Romon T. Berciano J. Figols J. Pazos A. (1998). 5-HT1B receptor binding in degenerative movement disorders. Brain Res. 790, 323–328 10.1016/S0006-8993(97)01566-7 9593971

[B27] Cavanagh J. T. Carson A. J. Sharpe M. Lawrie S. M. (2003). Psychological autopsy studies of suicide: a systematic review. Psychol. Med. 33, 395–405 10.1017/S0033291702006943 12701661

[B28] Cerqueira J. J. Catania C. Sotiropoulos I. Schubert M. Kalisch R. Almeida O. F. (2005a). Corticosteroid status influences the volume of the rat cingulate cortex – a magnetic resonance imaging study. J. Psychiatr. Res. 39, 451–460 10.1016/j.jpsychires.2005.01.003 15992553

[B29] Cerqueira J. J. Pego J. M. Taipa R. Bessa J. M. Almeida O. F. Sousa N. (2005b). Morphological correlates of corticosteroid-induced changes in prefrontal cortex-dependent behaviors. J. Neurosci. 25, 7792–7800 10.1523/JNEUROSCI.1598-05.2005 16120780 PMC6725252

[B30] Chao H. M. Sakai R. R. Ma L. Y. McEwen B. S. (1998). Adrenal steroid regulation of neurotrophic factor expression in the rat hippocampus. Endocrinology 139, 3112–3118 10.1210/en.139.7.3112 9645683

[B31] Chen B. Dowlatshahi D. MacQueen G. Wang J. F. Young T. L. (2001). Increased hippocampal BDNF immunoreactivity in subjects treated with antidepressant medication. Biol. Psychiatry 50, 260–265 10.1016/S0006-3223(01)01083-6 11522260

[B32] Choi S. H. Li Y. Parada L. F. Sisodia S. S. (2009). Regulation of hippocampal progenitor cell survival, proliferation and dendritic development by BDNF. Mol. Neurodegener. 4, 52 10.1186/1750-1326-4-52 20025751 PMC2806355

[B33] Christofides J. Bridel M. Egerton M. Mackay G. M. Forrest C. M. Stoy N. (2006). Blood 5-hydroxytryptamine, 5-hydroxyindoleacetic acid and melatonin levels in patients with either Huntington’s disease or chronic brain injury. J. Neurochem. 97, 1078–1088 10.1111/j.1471-4159.2006.03807.x 16573644

[B34] Ciammola A. Sassone J. Cannella M. Calza S. Poletti B. Frati L. (2007). Low brain-derived neurotrophic factor (BDNF) levels in serum of Huntington’s disease patients. Am. J. Med. Genet. B Neuropsychiatr. Genet. 144B, 574–577. 10.1002/ajmg.b.30501 17427191

[B35] Ciammola A. Sassone J. Colciago C. Mencacci N. E. Poletti B. Ciarmiello A. (2009). Aripiprazole in the treatment of Huntington’s disease: a case series. Neuropsychiatr. Dis. Treat. 5, 1–4 19557093 PMC2695210

[B36] Cirulli F. Berry A. Bonsignore L. T. Capone F. D’Andrea I. Aloe L. (2010). Early life influences on emotional reactivity: evidence that social enrichment has greater effects than handling on anxiety-like behaviors, neuroendocrine responses to stress and central BDNF levels. Neurosci. Biobehav. Rev. 34, 808–820 10.1016/j.neubiorev.2010.02.008 20171244

[B37] Cirulli F. Francia N. Berry A. Aloe L. Alleva E. Suomi S. J. (2009). Early life stress as a risk factor for mental health: role of neurotrophins from rodents to non-human primates. Neurosci. Biobehav. Rev. 33, 573–585 10.1016/j.neubiorev.2008.09.001 18817811 PMC2692357

[B38] Codori A. M. Slavney P. R. Rosenblatt A. Brandt J. (2004). Prevalence of major depression one year after predictive testing for Huntington’s disease. Genet. Test. 8, 114–119 10.1089/gte.2004.8.114 15345107

[B39] Cotman C. W. Berchtold N. C. (2002). Exercise: a behavioral intervention to enhance brain health and plasticity. Trends Neurosci. 25, 295–301 10.1016/S0166-2236(02)02143-4 12086747

[B40] Cotter D. R. Pariante C. M. Everall I. P. (2001). Glial cell abnormalities in major psychiatric disorders: the evidence and implications. Brain Res. Bull. 55, 585–595 10.1016/S0361-9230(01)00527-5 11576755

[B41] Cowansage K. K. LeDoux J. E. Monfils M. H. (2010). Brain-derived neurotrophic factor: a dynamic gatekeeper of neural plasticity. Curr. Mol. Pharmacol. 3, 12–29 10.2174/1874467211003010012 20030625

[B42] Crochemore C. Lu J. Wu Y. Liposits Z. Sousa N. Holsboer F. (2005). Direct targeting of hippocampal neurons for apoptosis by glucocorticoids is reversible by mineralocorticoid receptor activation. Mol. Psychiatry 10, 790–798 10.1038/sj.mp.4001679 15940303

[B43] Cross A. J. Reynolds G. P. Hewitt L. M. Slater P. (1986). Brain serotonin receptors in Huntington’s disease. Neurochem. Int. 9, 431–435 10.1016/0197-0186(86)90085-9 20493143

[B44] Czeh B. Simon M. (2005). Neuroplasticity and depression. Psychiatr. Hung. 20, 4–17 16389729

[B45] David G. Abbas N. Stevanin G. Durr A. Yvert G. Cancel G. (1997). Cloning of the SCA7 gene reveals a highly unstable CAG repeat expansion. Nat. Genet. 17, 65–70 10.1038/ng0997-65 9288099

[B46] Dawson L. A. Nguyen H. Q. Smith D. L. Schechter L. E. (2002). Effect of chronic fluoxetine and WAY-100635 treatment on serotonergic neurotransmission in the frontal cortex. J. Psychopharmacol. (Oxford) 16, 145–152 10.1177/026988110201600205 12095073

[B47] De Souza J. Jones L. A. Rickards H. (2010). Validation of self-report depression rating scales in Huntington’s disease. Mov. Disord. 25, 91–96 10.1002/mds.22837 19908314

[B48] Delgado P. L. Charney D. S. Price L. H. Aghajanian G. K. Landis H. Heninger G. R. (1990). Serotonin function and the mechanism of antidepressant action. Reversal of antidepressant-induced remission by rapid depletion of plasma tryptophan. Arch. Gen. Psychiatry 47, 411–418 10.1001/archpsyc.1990.01810170011002 2184795

[B49] DiFiglia M. Sapp E. Chase K. Schwarz C. Meloni A. Young C. (1995). Huntingtin is a cytoplasmic protein associated with vesicles in human and rat brain neurons. Neuron 14, 1075–1081 10.1016/0896-6273(95)90346-1 7748555

[B50] Drevets W. C. Frank E. Price J. C. Kupfer D. J. Holt D. Greer P. J. (1999). PET imaging of serotonin 1A receptor binding in depression. Biol. Psychiatry 46, 1375–1387 10.1016/S0006-3223(99)00189-4 10578452

[B51] Drevets W. C. Thase M. E. Moses-Kolko E. L. Price J. Frank E. Kupfer D. J. (2007). Serotonin-1A receptor imaging in recurrent depression: replication and literature review. Nucl. Med. Biol. 34, 865–877 10.1016/j.nucmedbio.2007.06.008 17921037 PMC2702715

[B52] Du X. Leang L. Mustafa T. Renoir T. Pang T. Y. Hannan A. J. (2012). Environmental enrichment rescues female-specific hyperactivity of the hypothalamic-pituitary-adrenal axis in a model of Huntington’s disease. Transl. Psychiatry 2, e133 10.1038/tp.2012.58 22760557 PMC3410631

[B53] Duff K. Paulsen J. S. Beglinger L. J. Langbehn D. R. Stout J. C. (2007). Psychiatric symptoms in Huntington’s disease before diagnosis: the predict-HD study. Biol. Psychiatry 62, 1341–1346 10.1016/j.biopsych.2006.11.034 17481592

[B54] Duman R. S. (2002). Pathophysiology of depression: the concept of synaptic plasticity. Eur. Psychiatry 17(Suppl. 3), 306–310 10.1016/S0924-9338(02)00654-5 15177086

[B55] Duman R. S. (2004). Role of neurotrophic factors in the etiology and treatment of mood disorders. Neuromolecular Med. 5, 11–25 10.1385/NMM:5:1:011 15001809

[B56] Duman R. S. Malberg J. Nakagawa S. D’Sa C. (2000). Neuronal plasticity and survival in mood disorders. Biol. Psychiatry 48, 732–739 10.1016/S0006-3223(00)00935-5 11063970

[B57] Duman R. S. Monteggia L. M. (2006). A neurotrophic model for stress-related mood disorders. Biol. Psychiatry 59, 1116–1127 10.1016/j.biopsych.2006.02.013 16631126

[B58] Dwivedi Y. Rizavi H. S. Pandey G. N. (2006). Antidepressants reverse corticosterone-mediated decrease in brain-derived neurotrophic factor expression: differential regulation of specific exons by antidepressants and corticosterone. Neuroscience 139, 1017–1029 10.1016/j.neuroscience.2005.12.058 16500030 PMC1513636

[B59] Erdeljan P. MacDonald J. F. Matthews S. G. (2001). Glucocorticoids and serotonin alter glucocorticoid receptor (GR) but not mineralocorticoid receptor (MR) mRNA levels in fetal mouse hippocampal neurons, in vitro. Brain Res. 896, 130–136 10.1016/S0006-8993(01)02075-3 11277981

[B60] Falkenberg V. R. Rajeevan M. S. (2010). Identification of a potential molecular link between the glucocorticoid and serotonergic signaling systems. J. Mol. Neurosci. 41, 322–327 10.1007/s12031-009-9320-6 20052562

[B61] Farrer L. A. (1986). Suicide and attempted suicide in Huntington disease: implications for preclinical testing of persons at risk. Am. J. Med. Genet. 24, 305–311 10.1002/ajmg.1320240211 2940862

[B62] Faure A. Höhn S. Von Hörsten S. Delatour B. Raber K. Le Blanc P. (2011). Altered emotional and motivational processing in the transgenic rat model for Huntington’s disease. Neurobiol. Learn. Mem. 95, 92–101 10.1016/j.nlm.2010.11.010 21111837

[B63] Focking M. Holker I. Trapp T. (2003). Chronic glucocorticoid receptor activation impairs CREB transcriptional activity in clonal neurons. Biochem. Biophys. Res. Commun. 304, 720–723 10.1016/S0006-291X(03)00665-X 12727214

[B64] Freeman M. P. (2009). Omega-3 fatty acids in major depressive disorder. J. Clin. Psychiatry 70(Suppl. 5), 7–11 10.4088/JCP.8157su1c.02 19909687

[B65] Froger N. Palazzo E. Boni C. Hanoun N. Saurini F. Joubert C. (2004). Neurochemical and behavioral alterations in glucocorticoid receptor-impaired transgenic mice after chronic mild stress. J. Neurosci. 24, 2787–2796 10.1523/JNEUROSCI.4132-03.2004 15028772 PMC6729531

[B66] Gargiulo M. Lejeune S. Tanguy M. L. Lahlou-Laforêt K. Faudet A. Cohen D. (2009). Long-term outcome of presymptomatic testing in Huntington disease. Eur. J. Hum. Genet. 17, 165–171 10.1038/ejhg.2008.146 18716614 PMC2986057

[B67] Gauthier L. R. Charrin B. C. Borrell-Pagès M. Dompierre J. P. Rangone H. Cordelières F. P. (2004). Huntingtin controls neurotrophic support and survival of neurons by enhancing BDNF vesicular transport along microtubules. Cell 118, 127–138 10.1016/j.cell.2004.06.018 15242649

[B68] Giralt A. Saavedra A. Carretón O. Arumí H. Tyebji S. Alberch J. (2013). PDE10 inhibition increases GluA1 and CREB phosphorylation and improves spatial and recognition memories in a Huntington’s disease mouse model. Hippocampus. [Epub ahead of print]. 10.1002/hipo.22128 23576401

[B69] Gorski J. A. Balogh S. A. Wehner J. M. Jones K. R. (2003). Learning deficits in forebrain-restricted brain-derived neurotrophic factor mutant mice. Neuroscience 121, 341–354 10.1016/S0306-4522(03)00426-3 14521993

[B70] Grote H. E. Bull N. D. Howard M. L. van Dellen A. Blakemore C. Bartlett P. F. (2005). Cognitive disorders and neurogenesis deficits in Huntington’s disease mice are rescued by fluoxetine. Eur. J. Neurosci. 22, 2081–2088 10.1111/j.1460-9568.2005.04365.x 16262645

[B71] Groves J. O. (2007). Is it time to reassess the BDNF hypothesis of depression? Mol. Psychiatry 12, 1079–1088 10.1038/sj.mp.4002075 17700574

[B72] Harada K. Yamaji T. Matsuoka N. (2008). Activation of the serotonin 5-HT2C receptor is involved in the enhanced anxiety in rats after single-prolonged stress. Pharmacol. Biochem. Behav. 89, 11–16 10.1016/j.pbb.2007.10.016 18067955

[B73] Harris E. C. Barraclough B. (1998). Excess mortality of mental disorder. Br. J. Psychiatry 173, 11–53 10.1192/bjp.173.1.11 9850203

[B74] Hasin D. S. Goodwin R. D. Stinson F. S. Grant B. F. (2005). Epidemiology of major depressive disorder: results from the National Epidemiologic Survey on Alcoholism and Related Conditions. Arch. Gen. Psychiatry 62, 1097–1106 10.1001/archpsyc.62.10.1097 16203955

[B75] Heisler L. K. Chu H. M. Brennan T. J. Danao J. A. Bajwa P. Parsons L. H. (1998). Elevated anxiety and antidepressant-like responses in serotonin 5-HT1A receptor mutant mice. Proc. Natl. Acad. Sci. U.S.A. 95, 15049–15054 10.1073/pnas.95.25.15049 9844013 PMC24573

[B76] Henn F. A. Vollmayr B. (2005). Stress models of depression: forming genetically vulnerable strains. Neurosci. Biobehav. Rev. 29, 799–804 10.1016/j.neubiorev.2005.03.019 15925700

[B77] Hery M. Semont A. Fache M. P. Faudon M. Hery F. (2000). The effects of serotonin on glucocorticoid receptor binding in rat raphe nuclei and hippocampal cells in culture. J. Neurochem. 74, 406–413 10.1046/j.1471-4159.2000.0740406.x 10617146

[B78] Hesen W. Joels M. (1996). Modulation of 5HT1A responsiveness in CA1 pyramidal neurons by in vivo activation of corticosteroid receptors. J. Neuroendocrinol. 8, 433–438 10.1046/j.1365-2826.1996.04724.x 8809673

[B79] Heuser I. J. Chase T. N. Mouradian M. M. (1991). The limbic-hypothalamic-pituitary-adrenal axis in Huntington’s disease. Biol. Psychiatry 30, 943–952 10.1016/0006-3223(91)90007-9 1660734

[B80] Ho A. K. Gilbert A. S. Mason S. L. Goodman A. O. Barker R. A. (2009). Health-related quality of life in Huntington’s disease: which factors matter most? Mov. Disord. 24, 574–578 10.1002/mds.22412 19097181

[B81] Holl A. K. Wilkinson L. Painold A. Holl E. M. Bonelli R. M. (2010). Combating depression in Huntington’s disease: effective antidepressive treatment with venlafaxine XR. Int. Clin. Psychopharmacol. 25, 46–50 10.1097/YIC.0b013e3283348018 19996754

[B82] Horowitz M. J. Field N. P. Zanko A. Donnelly E. F. Epstein C. Longo F. (2001). Psychological impact of news of genetic risk for Huntington disease. Am. J. Med. Genet. 103, 188–192 10.1002/ajmg.1538 11745989

[B83] Huang J. Strafaci J. A. Azmitia E. C. (1997). 5-HT1A receptor agonist reverses adrenalectomy-induced loss of granule neuronal morphology in the rat dentate gyrus. Neurochem. Res. 22, 1329–1337 10.1023/A:1022062921438 9355105

[B84] Inskip H. M. Harris E. C. Barraclough B. (1998). Lifetime risk of suicide for affective disorder, alcoholism and schizophrenia. Br. J. Psychiatry 172, 35–37 10.1192/bjp.172.1.35 9534829

[B85] Jacobs B. L. van Praag H. Gage F. H. (2000). Adult brain neurogenesis and psychiatry: a novel theory of depression. Mol. Psychiatry 5, 262–269 10.1038/sj.mp.4000712 10889528

[B86] Jensen P. Sorensen S. A. Fenger K. Bolwig T. G. (1993). A study of psychiatric morbidity in patients with Huntington’s disease, their relatives, and controls. Admissions to psychiatric hospitals in Denmark from 1969 to 1991. Br. J. Psychiatry 163, 790–797 10.1192/bjp.163.6.790 8306121

[B87] Jongen P. J. Renier W. O. Gabreels F. J. (1980). Seven cases of Huntington’s disease in childhood and levodopa induced improvement in the hypokinetic – rigid form. Clin. Neurol. Neurosurg. 82, 251–261 10.1016/0303-8467(80)90017-7 6165509

[B88] Julien C. L. Thompson J. C. Wild S. Yardumian P. Snowden J. S. Turner G. (2007). Psychiatric disorders in preclinical Huntington’s disease. J. Neurol. Neurosurg. Psychiatr. 78, 939–943 10.1136/jnnp.2006.103309 17178819 PMC2117854

[B89] Kakizuka A. (1997). Degenerative ataxias: genetics, pathogenesis and animal models. Curr. Opin. Neurol. 10, 285–290 10.1097/00019052-199708000-00002 9266151

[B90] Karege F. Vaudan G. Schwald M. Perroud N. La Harpe R. (2005). Neurotrophin levels in postmortem brains of suicide victims and the effects of antemortem diagnosis and psychotropic drugs. Brain Res. Mol. Brain Res. 136, 29–37 10.1016/j.molbrainres.2004.12.020 15893584

[B91] Kazantsev A. Preisinger E. Dranovsky A. Goldgaber D. Housman D. (1999). Insoluble detergent-resistant aggregates form between pathological and nonpathological lengths of polyglutamine in mammalian cells. Proc. Natl. Acad. Sci. U.S.A. 96, 11404–11409 10.1073/pnas.96.20.11404 10500189 PMC18046

[B92] Kazlauckas V. Pagnussat N. Mioranzza S. Kalinine E. Nunes F. Pettenuzzo L. (2011). Enriched environment effects on behavior, memory and BDNF in low and high exploratory mice. Physiol. Behav. 102, 475–480 10.1016/j.physbeh.2010.12.025 21236277

[B93] Kessing L. V. Willer I. S. Knorr U. (2011). Volume of the adrenal and pituitary glands in depression. Psychoneuroendocrinology 36, 19–27 10.1016/j.psyneuen.2010.05.007 20646833

[B94] Kessler R. C. Berglund P. Demler O. Jin R. Merikangas K. R. Walters E. E. (2005). Lifetime prevalence and age-of-onset distributions of DSM-IV disorders in the National Comorbidity Survey Replication. Arch. Gen. Psychiatry 62, 593–602 10.1001/archpsyc.62.6.617 15939837

[B95] Koponen E. Rantamaki T. Voikar V. Saarelainen T. MacDonald E. Castren E. (2005). Enhanced BDNF signaling is associated with an antidepressant-like behavioral response and changes in brain monoamines. Cell. Mol. Neurobiol. 25, 973–980 10.1007/s10571-005-8468-z 16392030 PMC11529533

[B96] Koshy B. T. Zoghbi H. Y. (1997). The CAG/polyglutamine tract diseases: gene products and molecular pathogenesis. Brain Pathol. 7, 927–942 10.1111/j.1750-3639.1997.tb00894.x 9217976 PMC8098410

[B97] Krogias C. Strassburger K. Eyding J. Gold R. Norra C. Juckel G. (2011). Depression in patients with Huntington disease correlates with alterations of the brain stem raphe depicted by transcranial sonography. J. Psychiatry Neurosci. 36, 187–194 10.1503/jpn.100067 21138658 PMC3080514

[B98] Krzyszton-Russjan J. Zielonka D. Jackiewicz J. Kusmirek S. Bubko I. Klimberg A. (2012). A study of molecular changes relating to energy metabolism and cellular stress in people with Huntington’s disease: looking for biomarkers. J. Bioenerg. Biomembr. 45, 71–85 23070563 10.1007/s10863-012-9479-3

[B99] Kusserow H. Davies B. Hörtnagl H. Voigt I. Stroh T. Bert B. (2004). Reduced anxiety-related behaviour in transgenic mice overexpressing serotonin 1A receptors. Brain Res. Mol. Brain Res. 129, 104–116 10.1016/j.molbrainres.2004.06.028 15469887

[B100] Lanfumey L. Mannoury La Cour C. Froger N. Hamon M. (2000). 5-HT-HPA interactions in two models of transgenic mice relevant to major depression. Neurochem. Res. 25, 1199–1206 10.1023/A:1007683810230 11059794

[B101] Larsson M. U. Luszcz M. A. Bui T. H. Wahlin T. B. (2006). Depression and suicidal ideation after predictive testing for Huntington’s disease: a two-year follow-up study. J. Genet. Couns. 15, 361–374 10.1007/s10897-006-9027-6 16967331

[B102] Leblhuber F. Peichl M. Neubauer C. Reisecker F. Steinparz F. X. Windhager E. (1995). Serum dehydroepiandrosterone and cortisol measurements in Huntington’s chorea. J. Neurol. Sci. 132, 76–79 10.1016/0022-510X(95)00114-H 8523035

[B103] Leroi I. O’Hearn E. Marsh L. Lyketsos C. G. Rosenblatt A. Ross C. A. (2002). Psychopathology in patients with degenerative cerebellar diseases: a comparison to Huntington’s disease. Am. J. Psychiatry 159, 1306–1314 10.1176/appi.ajp.159.8.1306 12153822

[B104] Lewin G. R. Barde Y. A. (1996). Physiology of the neurotrophins. Annu. Rev. Neurosci. 19, 289–317 10.1146/annurev.ne.19.030196.001445 8833445

[B105] Liu B. Zhang H. Xu C. Yang G. Tao J. Huang J. (2011). Neuroprotective effects of icariin on corticosterone-induced apoptosis in primary cultured rat hippocampal neurons. Brain Res. 1375, 59–67 10.1016/j.brainres.2010.12.053 21182828

[B106] Lopez J. F. Chalmers D. T. Little K. Y. Watson S. J. (1998). A.E. Bennett Research Award. Regulation of serotonin1A, glucocorticoid, and mineralocorticoid receptor in rat and human hippocampus: implications for the neurobiology of depression. Biol. Psychiatry 43, 547–573 10.1016/S0006-3223(97)00484-8 9564441

[B107] Lovestone S. Hodgson S. Sham P. Differ A. M. Levy R. (1996). Familial psychiatric presentation of Huntington’s disease. J. Med. Genet. 33, 128–131 10.1136/jmg.33.2.128 8929949 PMC1051838

[B108] Lucassen P. J. Meerlo P. Naylor A. S. van Dam A. M. Dayer A. G. Fuchs E. (2010). Regulation of adult neurogenesis by stress, sleep disruption, exercise and inflammation: implications for depression and antidepressant action. Eur. Neuropsychopharmacol. 20, 1–17 10.1016/j.euroneuro.2009.08.003 19748235

[B109] MacQueen G. M. Ramakrishnan K. Croll S. D. Siuciak J. A. Yu G. Young L. T. (2001). Performance of heterozygous brain-derived neurotrophic factor knockout mice on behavioral analogues of anxiety, nociception, and depression. Behav. Neurosci. 115, 1145–1153 10.1037/0735-7044.115.5.1145 11584927

[B110] Manji H. K. Quiroz J. A. Sporn J. Payne J. L. Denicoff K. Gray N. A. (2003). Enhancing neuronal plasticity and cellular resilience to develop novel, improved therapeutics for difficult-to-treat depression. Biol. Psychiatry 53, 707–742 10.1016/S0006-3223(03)00117-3 12706957

[B111] Markianos M. Panas M. Kalfakis N. Vassilopoulos D. (2004). Platelet monoamine oxidase activity in subjects tested for Huntington’s disease gene mutation. J. Neural Transm. 111, 475–483 10.1007/s00702-003-0103-x 15057517

[B112] McAllister A. K. (2001). Neurotrophins and neuronal differentiation in the central nervous system. Cell. Mol. Life Sci. 58, 1054–1060 10.1007/PL00000920 11529498 PMC11337387

[B113] McAllister A. K. Katz L. C. Lo D. C. (1999). Neurotrophins and synaptic plasticity. Annu. Rev. Neurosci. 22, 295–318 10.1146/annurev.neuro.22.1.295 10202541

[B114] Meaney M. J. Diorio J. Francis D. LaRocque S. O’Donnell D. Smythe J. W. (1994). Environmental regulation of the development of glucocorticoid receptor systems in the rat forebrain. The role of serotonin. Ann. N. Y. Acad. Sci. 746, 260–273; discussion 89–93. 10.1111/j.1749-6632.1994.tb39243.x 7825882

[B115] Messa C. Colombo C. Moresco R. M. Gobbo C. Galli L. Lucignani G. (2003). 5-HT(2A) receptor binding is reduced in drug-naive and unchanged in SSRI-responder depressed patients compared to healthy controls: a PET study. Psychopharmacology (Berl.) 167, 72–78 12632246 10.1007/s00213-002-1379-5

[B116] Meyer J. H. Ginovart N. Boovariwala A. Sagrati S. Hussey D. Garcia A. (2006). Elevated monoamine oxidase a levels in the brain: an explanation for the monoamine imbalance of major depression. Arch. Gen. Psychiatry 63, 1209–1216 10.1001/archpsyc.63.11.1209 17088501

[B117] Milnerwood A. J. Raymond L. A. (2010). Early synaptic pathophysiology in neurodegeneration: insights from Huntington’s disease. Trends Neurosci. 33, 513–523 10.1016/j.tins.2010.08.002 20850189

[B118] Molendijk M. L. Bus B. A. Spinhoven P. Penninx B. W. Kenis G. Prickaerts J. (2011). Serum levels of brain-derived neurotrophic factor in major depressive disorder: state-trait issues, clinical features and pharmacological treatment. Mol. Psychiatry 16, 1088–1095 10.1038/mp.2010.98 20856249 PMC3220395

[B119] Moller S. E. Honore P. Larsen O. B. (1983). Tryptophan and tyrosine ratios to neutral amino acids in endogenous depression. Relation to antidepressant response to amitriptyline and lithium + L-tryptophan. J. Affect. Disord. 5, 67–79 10.1016/0165-0327(83)90038-1 6220046

[B120] Monteggia L. M. Barrot M. Powell C. M. Berton O. Galanis V. Gemelli T. (2004). Essential role of brain-derived neurotrophic factor in adult hippocampal function. Proc. Natl. Acad. Sci. U.S.A. 101, 10827–10832 10.1073/pnas.0402141101 15249684 PMC490019

[B121] Montkowski A. Holsboer F. (1997). Intact spatial learning and memory in transgenic mice with reduced BDNF. Neuroreport 8, 779–782 10.1097/00001756-199702100-00040 9106766

[B122] Moses-Kolko E. L. Wisner K. L. Price J. C. Berga S. L. Drevets W. C. Hanusa B. H. (2008). Serotonin 1A receptor reductions in postpartum depression: a positron emission tomography study. Fertil. Steril. 89, 685–692 10.1016/j.fertnstert.2007.03.059 17543959 PMC2410091

[B123] Naarding P. Kremer H. P. Zitman F. G. (2001). Huntington’s disease: a review of the literature on prevalence and treatment of neuropsychiatric phenomena. Eur. Psychiatry 16, 439–445 10.1016/S0924-9338(01)00604-6 11777733

[B124] Nagappan G. Lu B. (2005). Activity-dependent modulation of the BDNF receptor TrkB: mechanisms and implications. Trends Neurosci. 28, 464–471 10.1016/j.tins.2005.07.003 16040136

[B125] Nakamura K. (2001). SCA17, a novel polyglutamine disease caused by the expansion of polyglutamine tracts in TATA-binding protein. Rinsho Shinkeigaku 41, 1123–1125 12235815

[B126] Nakamura K. Jeong S. Y. Uchihara T. Anno M. Nagashima K. Nagashima T. (2001). SCA17, a novel autosomal dominant cerebellar ataxia caused by an expanded polyglutamine in TATA-binding protein. Hum. Mol. Genet. 10, 1441–1448 10.1093/hmg/10.14.1441 11448935

[B127] Nance M. A. Myers R. H. (2001). Juvenile onset Huntington’s disease – clinical and research perspectives. Ment. Retard Dev. Disabil. Res. Rev. 7, 153–157 10.1002/mrdd.1022 11553930

[B128] Nehl C. Ready R. E. Hamilton J. Paulsen J. S. (2001). Effects of depression on working memory in presymptomatic Huntington’s disease. J. Neuropsychiatry Clin. Neurosci. 13, 342–346 10.1176/appi.neuropsych.13.3.342 11514640

[B129] Nibuya M. Nestler E. J. Duman R. S. (1996). Chronic antidepressant administration increases the expression of cAMP response element binding protein (CREB) in rat hippocampus. J. Neurosci. 16, 2365–2372 8601816 10.1523/JNEUROSCI.16-07-02365.1996 PMC6578518

[B130] Nithianantharajah J. Barkus C. Murphy M. Hannan A. J. (2008). Gene-environment interactions modulating cognitive function and molecular correlates of synaptic plasticity in Huntington’s disease transgenic mice. Neurobiol. Dis. 29, 490–504 10.1016/j.nbd.2007.11.006 18165017

[B131] Nithianantharajah J. Hannan A. J. (2012). Dysregulation of synaptic proteins, dendritic spine abnormalities and pathological plasticity of synapses as experience-dependent mediators of cognitive and psychiatric symptoms in Huntington’s disease. Neuroscience. [Epub ahead of print]. 10.1016/j.neuroscience.2012.05.043 22633949

[B132] Nucifora F. C. Jr. Sasaki M. Peters M. F. Huang H. Cooper J. K. Yamada M. (2001). Interference by Huntingtin and atrophin-1 with CBP-mediated transcription leading to cellular toxicity. Science 291, 2423–2428 10.1126/science.1056784 11264541

[B133] Numakawa T. Kumamaru E. Adachi N. Yagasaki Y. Izumi A. Kunugi H. (2009). Glucocorticoid receptor interaction with TrkB promotes BDNF-triggered PLC-gamma signaling for glutamate release via a glutamate transporter. Proc. Natl. Acad. Sci. U.S.A. 106, 647–652 10.1073/pnas.0800888106 19126684 PMC2626757

[B134] Ongur D. Drevets W. C. Price J. L. (1998). Glial reduction in the subgenual prefrontal cortex in mood disorders. Proc. Natl. Acad. Sci. U.S.A. 95, 13290–13295 10.1073/pnas.95.22.13290 9789081 PMC23786

[B135] Orvoen S. Pla P. Gardier A. M. Saudou F. David D. J. (2012). Huntington’s disease knock-in male mice show specific anxiety-like behaviour and altered neuronal maturation. Neurosci. Lett. 507, 127–132 10.1016/j.neulet.2011.11.063 22178857

[B136] Pang T. Y. Du X. Zajac M. S. Howard M. L. Hannan A. J. (2009). Altered serotonin receptor expression is associated with depression-related behavior in the R6/1 transgenic mouse model of Huntington’s disease. Hum. Mol. Genet. 18, 753–766 10.1093/hmg/ddn385 19008301

[B137] Pang T. Y. Stam N. C. Nithianantharajah J. Howard M. L. Hannan A. J. (2006). Differential effects of voluntary physical exercise on behavioral and brain-derived neurotrophic factor expression deficits in Huntington’s disease transgenic mice. Neuroscience 141, 569–584 10.1016/j.neuroscience.2006.04.013 16716524

[B138] Papadimitriou A. Priftis K. N. (2009). Regulation of the hypothalamic-pituitary-adrenal axis. Neuroimmunomodulation 16, 265–271 10.1159/000216184 19571587

[B139] Pariante C. M. Lightman S. L. (2008). The HPA axis in major depression: classical theories and new developments. Trends Neurosci. 31, 464–468 10.1016/j.tins.2008.06.006 18675469

[B140] Parks C. L. Robinson P. S. Sibille E. Shenk T. Toth M. (1998). Increased anxiety of mice lacking the serotonin1A receptor. Proc. Natl. Acad. Sci. U.S.A. 95, 10734–10739 10.1073/pnas.95.18.10734 9724773 PMC27964

[B141] Patel S. V. Tariot P. N. Asnis J. (1996). L-Deprenyl augmentation of fluoxetine in a patient with Huntington’s disease. Ann. Clin. Psychiatry 8, 23–26 10.3109/10401239609149087 8743645

[B142] Paulsen J. S. Nehl C. Hoth K. F. Kanz J. E. Benjamin M. Conybeare R. (2005). Depression and stages of Huntington’s disease. J. Neuropsychiatry Clin. Neurosci. 17, 496–502 10.1176/appi.neuropsych.17.4.496 16387989

[B143] Paulsen J. S. Ready R. E. Hamilton J. M. Mega M. S. Cummings J. L. (2001). Neuropsychiatric aspects of Huntington’s disease. J. Neurol. Neurosurg. Psychiatr. 71, 310–314 10.1136/jnnp.71.3.310 11511702 PMC1737562

[B144] Pencea V. Bingaman K. D. Wiegand S. J. Luskin M. B. (2001). Infusion of brain-derived neurotrophic factor into the lateral ventricle of the adult rat leads to new neurons in the parenchyma of the striatum, septum, thalamus, and hypothalamus. J. Neurosci. 21, 6706–6717 11517260 10.1523/JNEUROSCI.21-17-06706.2001 PMC6763082

[B145] Peng Q. Masuda N. Jiang M. Li Q. Zhao M. Ross C. A. (2008). The antidepressant sertraline improves the phenotype, promotes neurogenesis and increases BDNF levels in the R6/2 Huntington’s disease mouse model. Exp. Neurol. 210, 154–163 10.1016/j.expneurol.2007.10.015 18096160 PMC2278120

[B146] Petersen A. Wortwein G. Gruber S. H. Mathe A. A. (2008). Escitalopram reduces increased hippocampal cytogenesis in a genetic rat depression model. Neurosci. Lett. 436, 305–308 10.1016/j.neulet.2008.03.035 18406530

[B147] Pizarro J. M. Lumley L. A. Medina W. Robison C. L. Chang W. E. Alagappan A. (2004). Acute social defeat reduces neurotrophin expression in brain cortical and subcortical areas in mice. Brain Res. 1025, 10–20 10.1016/j.brainres.2004.06.085 15464739

[B148] Pouladi M. A. Graham R. K. Karasinska J. M. Xie Y. Santos R. D. Petersén A. (2009). Prevention of depressive behaviour in the YAC128 mouse model of Huntington disease by mutation at residue 586 of huntingtin. Brain 132, 919–932 10.1093/brain/awp006 19224899

[B149] Quintana J. (1992). Platelet serotonin and plasma tryptophan decreases in endogenous depression. Clinical, therapeutic, and biological correlations. J. Affect. Disord. 24, 55–62 10.1016/0165-0327(92)90019-3 1541767

[B150] Rajkowska G. (2002). Cell pathology in mood disorders. Semin. Clin. Neuropsychiatry 7, 281–292 10.1053/scnp.2002.35228 12382210

[B151] Ramboz S. Oosting R. Amara D. A. Kung H. F. Blier P. Mendelsohn M. (1998). Serotonin receptor 1A knockout: an animal model of anxiety-related disorder. Proc. Natl. Acad. Sci. U.S.A. 95, 14476–14481 10.1073/pnas.95.24.14476 9826725 PMC24398

[B152] Raymond L. A. Andre V. M. Cepeda C. Gladding C. M. Milnerwood A. J. Levine M. S. (2011). Pathophysiology of Huntington’s disease: time-dependent alterations in synaptic and receptor function. Neuroscience 198, 252–273 10.1016/j.neuroscience.2011.08.052 21907762 PMC3221774

[B153] Renoir T. Chevarin C. Lanfumey L. Hannan A. J. (2011a). Effect of enhanced voluntary physical exercise on brain levels of monoamines in Huntington disease mice. PLoS Curr. 3:RRN1281 10.1371/currents.RRN1281 22266953 PMC3208413

[B154] Renoir T. Zajac M. S. Du X. Pang T. Y. Leang L. Chevarin C. (2011b). Sexually dimorphic serotonergic dysfunction in a mouse model of Huntington’s disease and depression. PLoS ONE 6:e22133 10.1371/journal.pone.0022133 21760962 PMC3132782

[B155] Renoir T. Pang T. Y. Mo C. Chan G. Chevarin C. Lanfumey L. (2013). Differential effects of early environmental enrichment on emotionality related behaviours in Huntington’s disease transgenic mice. J. Physiol. (Lond.) 591, 41–55 10.1113/jphysiol.2012.239798 23045340 PMC3630770

[B156] Renoir T. Pang T. Y. Zajac M. S. Chan G. Du X. Leang L. (2012). Treatment of depressive-like behaviour in Huntington’s disease mice by chronic sertraline and exercise. Br. J. Pharmacol. 165, 1375–1389 10.1111/j.1476-5381.2011.01567.x 21718306 PMC3372723

[B157] Richards G. Messer J. Waldvogel H. J. Gibbons H. M. Dragunow M. Faull R. L. (2011). Up-regulation of the isoenzymes MAO-A and MAO-B in the human basal ganglia and pons in Huntington’s disease revealed by quantitative enzyme radioautography. Brain Res. 1370, 204–214 10.1016/j.brainres.2010.11.020 21075085

[B158] Rickards H. De Souza J. Crooks J. van Walsem M. R. van Duijn E. Landwehrmeyer B. (2011). Discriminant analysis of beck depression inventory and hamilton rating scale for depression in Huntington’s disease. J. Neuropsychiatry Clin. Neurosci. 23, 399–402 10.1176/appi.neuropsych.23.4.399 22231310

[B159] Robitaille Y. Lopes-Cendes I. Becher M. Rouleau G. Clark A. W. (1997). The neuropathology of CAG repeat diseases: review and update of genetic and molecular features. Brain Pathol. 7, 901–926 10.1111/j.1750-3639.1997.tb00893.x 9217975 PMC8098401

[B160] Ross C. A. (1997). Intranuclear neuronal inclusions: a common pathogenic mechanism for glutamine-repeat neurodegenerative diseases? Neuron 19, 1147–1150 10.1016/S0896-6273(00)80405-5 9427237

[B161] Ross C. A. Tabrizi S. J. (2011). Huntington’s disease: from molecular pathogenesis to clinical treatment. Lancet Neurol. 10, 83–98 10.1016/S1474-4422(10)70245-3 21163446

[B162] Rossi D. V. Burke T. F. McCasland M. Hensler J. G. (2008). Serotonin-1A receptor function in the dorsal raphe nucleus following chronic administration of the selective serotonin reuptake inhibitor sertraline. J. Neurochem. 105, 1091–1099 10.1111/j.1471-4159.2007.05201.x 18182050

[B163] Rowe K. C. Paulsen J. S. Langbehn D. R. Wang C. Mills J. Beglinger L. J. (2012). Patterns of serotonergic antidepressant usage in prodromal Huntington disease. Psychiatry Res. 196, 309–314 10.1016/j.psychres.2011.09.005 22397915 PMC3763706

[B164] Roy A. De Jong J. Linnoila M. (1989). Cerebrospinal fluid monoamine metabolites and suicidal behavior in depressed patients. A 5-year follow-up study. Arch. Gen. Psychiatry 46, 609–612 10.1001/archpsyc.1989.01810070035005 2472124

[B165] Saarelainen T. Hendolin P. Lucas G. Koponen E. Sairanen M. MacDonald E. (2003). Activation of the TrkB neurotrophin receptor is induced by antidepressant drugs and is required for antidepressant-induced behavioral effects. J. Neurosci. 23, 349–357 12514234 10.1523/JNEUROSCI.23-01-00349.2003 PMC6742146

[B166] Sackley C. Hoppitt T. J. Calvert M. Gill P. Eaton B. Yao G. (2011). Huntington’s disease: current epidemiology and pharmacological management in UK primary care. Neuroepidemiology 37, 216–221 10.1159/000331912 22133668

[B167] Sapolsky R. M. (1986). Glucocorticoid toxicity in the hippocampus. Temporal aspects of synergy with kainic acid. Neuroendocrinology 43, 440–444 10.1159/000124561 3736786

[B168] Sapolsky R. M. Uno H. Rebert C. S. Finch C. E. (1990). Hippocampal damage associated with prolonged glucocorticoid exposure in primates. J. Neurosci. 10, 2897–2902 2398367 10.1523/JNEUROSCI.10-09-02897.1990 PMC6570248

[B169] Schaaf M. J. de Jong J. de Kloet E. R. Vreugdenhil E. (1998). Downregulation of BDNF mRNA and protein in the rat hippocampus by corticosterone. Brain Res. 813, 112–120 10.1016/S0006-8993(98)01010-5 9824681

[B170] Schaaf M. J. Hoetelmans R. W. de Kloet E. R. Vreugdenhil E. (1997). Corticosterone regulates expression of BDNF and trkB but not NT-3 and trkC mRNA in the rat hippocampus. J. Neurosci. Res. 48, 334–341 10.1002/(SICI)1097-4547(19970515)48:4<334::AID-JNR5>3.0.CO;2-C 9169859

[B171] Schmitz-Hübsch T. Coudert M. Tezenas du Montcel S. Giunti P. Labrum R. Dürr A. (2011). Depression comorbidity in spinocerebellar ataxia. Mov. Disord. 26, 870–876 10.1002/mds.23698 21437988

[B172] Shimizu E. Hashimoto K. Okamura N. Koike K. Komatsu N. Kumakiri C. (2003). Alterations of serum levels of brain-derived neurotrophic factor (BDNF) in depressed patients with or without antidepressants. Biol. Psychiatry 54, 70–75 10.1016/S0006-3223(03)00181-1 12842310

[B173] Shiwach R. (1994). Psychopathology in Huntington’s disease patients. Acta Psychiatr. Scand. 90, 241–246 10.1111/j.1600-0447.1994.tb01587.x 7831992

[B174] Shiwach R. S. Norbury C. G. (1994). A controlled psychiatric study of individuals at risk for Huntington’s disease. Br. J. Psychiatry 165, 500–505 10.1192/bjp.165.4.500 7804665

[B175] Siuciak J. A. Lewis D. R. Wiegand S. J. Lindsay R. M. (1997). Antidepressant-like effect of brain-derived neurotrophic factor (BDNF). Pharmacol. Biochem. Behav. 56, 131–137 10.1016/S0091-3057(96)00169-4 8981620

[B176] Smith M. A. Makino S. Kvetnansky R. Post R. M. (1995). Effects of stress on neurotrophic factor expression in the rat brain. Ann. N. Y. Acad. Sci. 771, 234–239 10.1111/j.1749-6632.1995.tb44684.x 8597402

[B177] Smith M. M. Mills J. A. Epping E. A. Westervelt H. J. Paulsen J. S. (2012). Depressive symptom severity is related to poorer cognitive performance in prodromal Huntington disease. Neuropsychology 26, 664–669 10.1037/a0029218 22846033 PMC3806339

[B178] Spires T. L. Grote H. E. Varshney N. K. Cordery P. M. van Dellen A. Blakemore C. (2004). Environmental enrichment rescues protein deficits in a mouse model of Huntington’s disease, indicating a possible disease mechanism. J. Neurosci. 24, 2270–2276 10.1523/JNEUROSCI.1658-03.2004 14999077 PMC6730435

[B179] Squitieri F. Cannella M. Piorcellini A. Brusa L. Simonelli M. Ruggieri S. (2001). Short-term effects of olanzapine in Huntington disease. Neuropsychiatry Neuropsychol. Behav. Neurol. 14, 69–72 11234911

[B180] Stetler C. Miller G. E. (2011). Depression and hypothalamic-pituitary-adrenal activation: a quantitative summary of four decades of research. Psychosom. Med. 73, 114–126 10.1097/PSY.0b013e31820ad12b 21257974

[B181] Stockmeier C. A. Shapiro L. A. Dilley G. E. Kolli T. N. Friedman L. Rajkowska G. (1998). Increase in serotonin-1A autoreceptors in the midbrain of suicide victims with major depression-postmortem evidence for decreased serotonin activity. J. Neurosci. 18, 7394–7401 9736659 10.1523/JNEUROSCI.18-18-07394.1998 PMC6793229

[B182] Strand A. D. Baquet Z. C. Aragaki A. K. Holmans P. Yang L. Cleren C. (2007). Expression profiling of Huntington’s disease models suggests that brain-derived neurotrophic factor depletion plays a major role in striatal degeneration. J. Neurosci. 27, 11758–11768 10.1523/JNEUROSCI.2461-07.2007 17959817 PMC6673215

[B183] Sullivan F. R. Bird E. D. Alpay M. Cha J. H. (2001). Remotivation therapy and Huntington’s disease. J. Neurosci. Nurs. 33, 136–142 10.1097/01376517-200106000-00005 11413658

[B184] Sun H. Zhang J. Zhang L. Liu H. Zhu H. Yang Y. (2010). Environmental enrichment influences BDNF and NR1 levels in the hippocampus and restores cognitive impairment in chronic cerebral hypoperfused rats. Curr. Neurovasc. Res. 7, 268–280 10.2174/156720210793180819 20854252

[B185] Szigethy E. Conwell Y. Forbes N. T. Cox C. Caine E. D. (1994). Adrenal weight and morphology in victims of completed suicide. Biol. Psychiatry 36, 374–380 10.1016/0006-3223(94)91212-2 7803598

[B186] Tabuchi A. (2008). Synaptic plasticity-regulated gene expression: a key event in the long-lasting changes of neuronal function. Biol. Pharm. Bull. 31, 327–335 10.1248/bpb.31.327 18310887

[B187] The Huntington’s Disease Collaborative Research Group (1993). A novel gene containing a trinucleotide repeat that is expanded and unstable on Huntington’s disease chromosomes. The Huntington’s Disease Collaborative Research Group. Cell 72, 971–983 10.1016/0092-8674(93)90585-E 8458085

[B188] Thoenen H. (2000). Neurotrophins and activity-dependent plasticity. Prog. Brain Res. 128, 183–191 10.1016/S0079-6123(00)28016-3 11105678

[B189] Trembath M. K. Horton Z. A. Tippett L. Hogg V. Collins V. R. Churchyard A. (2010). A retrospective study of the impact of lifestyle on age at onset of Huntington disease. Mov. Disord. 25, 1444–1450 10.1002/mds.23108 20629137

[B190] Urani A. Chourbaji S. Gass P. (2005). Mutant mouse models of depression: candidate genes and current mouse lines. Neurosci. Biobehav. Rev. 29, 805–828 10.1016/j.neubiorev.2005.03.020 15925701

[B191] Vaidya V. A. Duman R. S. (2001). Depresssion – emerging insights from neurobiology. Br. Med. Bull. 57, 61–79 10.1093/bmb/57.1.61 11719924

[B192] van Dellen A. Blakemore C. Deacon R. York D. Hannan A. J. (2000). Delaying the onset of Huntington’s in mice. Nature 404, 721–722 10.1038/35008142 10783874

[B193] van Duijn E. Selis M. A. Giltay E. J. Zitman F. G. Roos R. A. van Pelt H. (2010). Hypothalamic-pituitary-adrenal axis functioning in Huntington’s disease mutation carriers compared with mutation-negative first-degree controls. Brain Res. Bull. 83, 232–237 10.1016/j.brainresbull.2010.08.006 20713132

[B194] van der Burg J. M. Bjorkqvist M. Brundin P. (2009). Beyond the brain: widespread pathology in Huntington’s disease. Lancet Neurol. 8, 765–774 10.1016/S1474-4422(09)70178-4 19608102

[B195] van Duijn E. Kingma E. M. Timman R. Zitman F. G. Tibben A. Roos R. A. (2008). Cross-sectional study on prevalences of psychiatric disorders in mutation carriers of Huntington’s disease compared with mutation-negative first-degree relatives. J. Clin. Psychiatry 69, 1804–1810 10.4088/JCP.v69n1116 19026253

[B196] van Duijn E. Kingma E. M. van der Mast R. C. (2007). Psychopathology in verified Huntington’s disease gene carriers. J. Neuropsychiatry Clin. Neurosci. 19, 441–448 10.1176/appi.neuropsych.19.4.441 18070848

[B197] van Praag H. M. Plutchik R. (1984). Depression type and depression severity in relation to risk of violent suicide attempt. Psychiatry Res. 12, 333–338 10.1016/0165-1781(84)90049-0 6209740

[B198] Vassos E. Panas M. Kladi A. Vassilopoulos D. (2008). Effect of CAG repeat length on psychiatric disorders in Huntington’s disease. J. Psychiatr. Res. 42, 544–549 10.1016/j.jpsychires.2007.05.008 17610899

[B199] Venna V. R. Deplanque D. Allet C. Belarbi K. Hamdane M. Bordet R. (2009). PUFA induce antidepressant-like effects in parallel to structural and molecular changes in the hippocampus. Psychoneuroendocrinology 34, 199–211 10.1016/j.psyneuen.2008.08.025 18848400

[B200] Waeber C. Palacios J. M. (1989). Serotonin-1 receptor binding sites in the human basal ganglia are decreased in Huntington’s chorea but not in Parkinson’s disease: a quantitative in vitro autoradiography study. Neuroscience 32, 337–347 10.1016/0306-4522(89)90082-1 2531301

[B201] Walter U. Prudente-Morrissey L. Herpertz S. C. Benecke R. Hoeppner J. (2007). Relationship of brainstem raphe echogenicity and clinical findings in depressive states. Psychiatry Res. 155, 67–73 10.1016/j.pscychresns.2006.12.001 17391931

[B202] Wang H. T. Han F. Shi Y. X. (2009). Activity of the 5-HT1A receptor is involved in the alteration of glucocorticoid receptor in hippocampus and corticotropin-releasing factor in hypothalamus in SPS rats. Int. J. Mol. Med. 24, 227–231 19578795 10.3892/ijmm_00000225

[B203] Weigell-Weber M. Schmid W. Spiegel R. (1996). Psychiatric symptoms and CAG expansion in Huntington’s disease. Am. J. Med. Genet. 67, 53–57 10.1002/(SICI)1096-8628(19960216)67:1<53::AIDAJMG9>3.0.CO;2-T 8678115

[B204] Wester P. Bergstrom U. Eriksson A. Gezelius C. Hardy J. Winblad B. (1990). Ventricular cerebrospinal fluid monoamine transmitter and metabolite concentrations reflect human brain neurochemistry in autopsy cases. J. Neurochem. 54, 1148–1156 10.1111/j.1471-4159.1990.tb01942.x 1968956

[B205] Wong E. Y. Herbert J. (2006). Raised circulating corticosterone inhibits neuronal differentiation of progenitor cells in the adult hippocampus. Neuroscience 137, 83–92 10.1016/j.neuroscience.2005.08.073 16289354 PMC2651634

[B206] Woolley C. S. Gould E. McEwen B. S. (1990). Exposure to excess glucocorticoids alters dendritic morphology of adult hippocampal pyramidal neurons. Brain Res. 531, 225–231 10.1016/0006-8993(90)90778-A 1705153

[B207] Yohrling I. G. Jiang G. C. DeJohn M. M. Robertson D. J. Vrana K. E. Cha J. H. (2002). Inhibition of tryptophan hydroxylase activity and decreased 5-HT1A receptor binding in a mouse model of Huntington’s disease. J. Neurochem. 82, 1416–1423 10.1046/j.1471-4159.2002.01084.x 12354289

[B208] Yoshida T. Ishikawa M. Niitsu T. Nakazato M. Watanabe H. Shiraishi T. (2012). Decreased serum levels of mature brain-derived neurotrophic factor (BDNF), but not its precursor proBDNF, in patients with major depressive disorder. PLoS ONE 7:e42676 10.1371/journal.pone.0042676 22880079 PMC3411809

[B209] Yoshimura R. Umene-Nakano W. Hoshuyama T. Ikenouchi-Sugita A. Hori H. Katsuki A. (2010). Plasma levels of brain-derived neurotrophic factor and interleukin-6 in patients with dysthymic disorder: comparison with age- and sex-matched major depressed patients and healthy controls. Hum. Psychopharmacol. 25, 566–569 10.1002/hup.1155 21312291

[B210] Zajac M. S. Pang T.Y. Wong N. Weinrich B. Leang L.S. Craig J. M. (2010). Wheel running and environmental enrichment differentially modify exon-specific BDNF expression in the hippocampus of wild-type and pre-motor symptomatic male and female Huntington’s disease mice. Hippocampus 20, 621–636 10.1002/hipo.20658 19499586

[B211] Zielonka D. Marinus J. Roos R. A. De Michele G. Di Donato S. Putter H. (2012). The influence of gender on phenotype and disease progression in patients with Huntington’s disease. Parkinsonism Relat. Disord. 19, 192–197 10.1016/j.parkreldis.2012.09.012 23102616

[B212] Zigova T. Pencea V. Wiegand S. J. Luskin M. B. (1998). Intraventricular administration of BDNF increases the number of newly generated neurons in the adult olfactory bulb. Mol. Cell. Neurosci. 11, 234–245 10.1006/mcne.1998.0684 9675054

[B213] Zuccato C. Ciammola A. Rigamonti D. Leavitt B. R. Goffredo D. Conti L. (2001). Loss of huntingtin-mediated BDNF gene transcription in Huntington’s disease. Science 293, 493–498 10.1126/science.1059581 11408619

[B214] Zwilling D. Huang S. Y. Sathyasaikumar K. V. Notarangelo F. M. Guidetti P. Wu H. Q. (2011). Kynurenine 3-monooxygenase inhibition in blood ameliorates neurodegeneration. Cell 145, 863–874 10.1016/j.cell.2011.05.020 21640374 PMC3118409

